# Preconditioned microbial communities in electrochemical sensing: initial assessment of detection capabilities and durability

**DOI:** 10.1128/aem.01151-25

**Published:** 2025-08-21

**Authors:** Yashawini Phriya Rauichandran, Kai Ling Yu, Mohd Nur Ikhmal Salehmin, Hassan Mohamed, Halimah Badioze Zaman, Samet Şahin, Eileen H. Yu, Ahmad Razi Othman, Wei Lun Ang, Swee Su Lim

**Affiliations:** 1 Fuel Cell Institute, Universiti Kebangsaan Malaysia 61775, Bangi, Selangor, Malaysia; 2 School of Engineering, Faculty of Engineering and Technology, Sunway University, Bandar Sunway, Selangor Darul Ehsan, Malaysia; 3 Tan Sri Leo Moggie Distinguished Chair in Energy Informatics, Institute of Informatics and Computing in Energy (IICE), Universiti Tenaga Nasional (UNITEN), Jalan IKRAM UNITEN 65292, Kajang, Selangor, Malaysia; 4 National Nanotechnology Center (NNC), Ministry of Science, Technology, and Innovation (MOSTI), Putrajaya, Malaysia; 5 Institute of Sustainable Energy, Universiti Tenaga Nasional (UNITEN), Jalan IKRAM UNITEN 65292, Kajang, Selangor, Malaysia; 6 School of Engineering, Lancaster University, Lancaster, United Kingdom; 7 School of Chemistry and Chemical Engineering, University of Southampton 7423, Southampton, United Kingdom; 8 Department of Chemical and Process Engineering, Faculty of Engineering and Built Environment, Universiti Kebangsaan Malaysia 61775, Bangi, Selangor, Malaysia; Washington University in St. Louis, St. Louis, Missouri, USA

**Keywords:** microbial electrochemical sensor, pre-enriched microbial cultures, water quality monitoring, *Geobacter *and SRB, toxicant testing

## Abstract

This study explores the role of pre-colonized microbial cultures in enhancing the long-term effectiveness of microbial electrochemical sensors for water quality monitoring. Microbial electrochemical sensors rely on specific functional microorganisms to detect and signal changes in environmental water quality. Pre-colonization of these cultures on the sensor’s electrode can promote sustained detection sensitivity. This study investigates how specific microbial groups—*Geobacter* species, sulfate-reducing bacteria (SRB), and acetogen microbes—affect sensor performance when initially enriched and transferred to the biosensor. In the pre-enrichment phase, microbes were incubated in three defined media: *Geobacter* medium, SRB medium, and acetogen medium. Each culture was cycled through three 48 h incubation periods to establish dominant microbial populations and then introduced to independent biosensors. A control sensor was seeded with natural inoculum from Tasek Kejuruteraan UKM. The results showed that *Geobacter*-enriched biosensors quickly generated strong electrical signals by oxidizing substrates at the anode, marking them as the most effective at facilitating electron transfer. SRB-enriched sensors produced negative signals, as SRB consumed electrons and thrived at the cathode. *Acetogen*-enriched biosensors exhibited slower, indirect electron transfer, with lower electrochemical activity. In contrast, the control sensor displayed only minimal increases in signal strength over time. The *Geobacter*-enriched biosensor, which achieved a significant current drop from 0.478 mA to 0.093 mA (an ~80.5% decrease) upon pollutant exposure, demonstrated the fastest response to rising pollutant levels, followed by SRB, acetogen, and the control. These findings emphasize the importance of starting with targeted microbial populations to optimize biosensor functionality for environmental monitoring applications.

**IMPORTANCE:**

Microbial electrochemical sensors are widely recognized as effective tools for environmental monitoring and water quality assessment. Numerous studies have explored the enrichment and adaptation of microbial communities in various environmental conditions, focusing on their interactions, survival, and metabolic performance. However, a critical gap remains largely overlooked—specifically, the importance of the biosensor start-up procedure and the selection of initial microbial populations. The presence of specific electrogenic bacteria at the sensing terminal during start-up plays a vital role in initiating and sustaining biosensor functionality. In this study, we aim to address this gap by not only examining the performance of the biosensor system itself but also emphasizing the role of pre-enriched microbial communities. Our approach focuses on building a healthy, functional, and responsive biosensing platform by optimizing microbial colonization from the onset.

## INTRODUCTION

The industrial revolution has been a significant contributor to environmental pollution. Heavy metals and organic compounds are among the most common pollutants hugely dispersed across the environment, particularly in the water, where they cause significant risks to aquatic ecosystems and animal health (1). Thus, monitoring water quality is a fundamental aspect of environmental protection and public health improvement, particularly in wastewater treatment systems. These systems often rely on biological processes where microorganisms break down organic and inorganic pollutants, converting them into less harmful substances (2). As such, maintaining the microbial health of these systems is vital for ensuring optimal treatment efficiency. However, traditional water quality monitoring methods are often plagued by limitations, including being labor-intensive, time-consuming, and prone to delays and sampling gaps (3). These conventional approaches involve manually collecting water samples for laboratory analysis of parameters such as pH, dissolved oxygen, and chemical constituents. Additionally, the common methods used for pollutant detection include high-performance liquid chromatography (HPLC), gas chromatography (GC), and organic acid (OA) test kits, involve manual sample handling, and rely on a spectrophotometer for the final analysis (4). These issues hinder the ability to detect real-time fluctuations or irregularities in water quality.

Real-time water quality monitoring has emerged as a solution to these challenges, offering immediate and continuous data to address the dynamic nature of wastewater treatment processes (5). This approach enables the early detection of issues such as equipment malfunctions or pollutant spikes, enhances operational efficiency, reduces costs, and minimizes the risk of outbreaks by maintaining water safety standards. Additionally, real-time monitoring plays a pivotal role in protecting ecosystems by preventing the release of harmful compounds into aquatic environments. These capabilities make real-time monitoring an indispensable tool for promoting sustainable practices and improving decision-making in water and wastewater management (6).

Among the technologies, biosensors have emerged as a key tool for the efficient detection of contaminants with high specificity and high sensitivity. Based on their biorecognition principle, biosensors can be classified into catalytic and non-catalytic biosensors. Catalytic biosensors, including microbes, tissues, enzymes, and whole cells, generate new biochemical reaction products, whereas non-catalytic ones, such as antibodies, cell receptors, and nucleic acids, detect analytes through irreversible analyte binding without producing new products (7, 8).

Biosensors have evolved through three main generations, with each improving the integration of biorecognition elements with transducers. The first introduced by Clark in 1956 relied on diffusion-based detection for oxygen sensing (9) and was later adapted for glucose monitoring purposes. The second added nanomaterials and mediators to boost efficiency (10), whereas the third enabled direct electron transfer, improving repeatability and reducing costs (11).

A key advancement was Liedberg’s introduction of surface plasmon resonance (SPR), followed by the first pen-sized glucose biosensor (12). Although antibody-based sensors offer faster detection and high sensitivity, they require expensive costs and controlled conditions, causing them to be less suitable for long-term environmental monitoring. Similarly, enzyme-based biosensors give fast reaction, but the performance can be affected by several factors, including temperature and pH fluctuations (7, 8). Microbial-based sensors, on the other hand, are able to adapt and replicate, making them well-suited for long-term applications.

In recent years, microbial electrochemical sensors have gained attention as a promising solution for real-time water quality monitoring (13, 14). Advancements in this technology have led to an improved ability to process both simple and complex wastewater, with electroactive microbes playing the main role in degrading the organic pollutants and aiding in electron transfer (15, 16). Moreover, toxicant biosensors had also been explored via microbial electrochemical sensors for the detection of heavy metals, antibiotics, and organic pollutants, with studies showing their microbial sensors can recover functionality after pollutant levels decline (17). These innovative sensors are low-cost, are easy to maintain, and utilize electrochemically active microbes to convert biochemical reactions directly into detectable electrical signals. This approach provides a responsive, real-time monitoring capability by following metabolic mechanisms that relate directly to pollutant concentrations. Despite the promise of microbial electrochemical sensors for real-time water quality monitoring, their practical applications are hindered by significant challenges. Their performance is highly susceptible to environmental fluctuations, such as changes in pH, temperature, and salinity, which may favor non-target microbes. This would lead to inconsistent or delayed responses (3). A critical research gap remains in developing strategies to ensure the establishment of robust microbial communities capable of delivering consistent and reliable performance. Addressing this limitation requires the stabilization and optimization of electroactive microbial cultures to improve biosensor efficiency.

One potential solution is to introduce pre-enriched, functional microbial cultures that can initially colonize the sensor and form a stable, effective microbial community. However, this approach remains underexplored, highlighting the need for further study into its potential to enhance sensor performance. This study hypothesizes that these pre-enriched microbial cultures will improve biosensor performance by promoting a stable and efficient community of functional microbes from the outset. The study aims to investigate how these pre-enriched cultures, specifically strains known for electrochemical activity (e.g., *Geobacter *sp. and sulfate-reducing bacteria), affect biosensor sensitivity, response time, and robustness. This approach could enhance the practical applications of microbial electrochemical sensors, contributing to advancements in water quality monitoring technologies for the treatment industry.

## MATERIALS AND METHODS

### Medium preparation and culture pre-enrichment

Three distinct enrichment media were prepared according to specifications outlined in Table 1. Each medium was distributed into 100 mL serum bottles, each containing 50 mL of medium. Bottles were sealed with aluminum caps and butyl rubber septa before being autoclaved at 121°C for a minimum of 15 min, following standard sterilization protocols.

**TABLE 1:** Composition and preparation of enrichment media for *Acetogen*, Sulfate-Reducing, and *Geobacter* cultures^*a*^

Acetogen medium		Postgate’s medium B for sulfate-reducing bacteria		*Geobacter* medium	
Chemicals	Weight^*b*^	Chemicals	Weight^*b*^	Chemicals	Weight^*b*^
NaHCO_3_	2.4 g	MgSO_4_·7H_2_O	2.0 g	Ferric citrate	13.7 g
NH_4_Cl	0.2 g	NH_4_Cl	1.0 g	Sodium acetate	2.5 g
Yeast extract	0.2 g	CaSO_4_	1.0 g	NaHCO_3_	2.5 g
Stock salts solution #1	40.0 mL	Yeast extract	1.0 g	NH_4_Cl	1.5 g
Potassium phosphate buffer	20.0 mL	KH_2_PO_4_	0.5 g	NaH_2_PO_4_	0.6 g
Clarified rumen fluid	20.0 mL	FeSO_4_·7H_2_O	0.5 g	KCl	0.1 g
Stock salts solution #2	4.0 mL	Ascorbic acid	0.1 g	Na_2_WO_4_·2H_2_O	0.25 mg
Trace minerals solution	4.0 mL	Thioglycolic acid	0.1 g	Trace elements solution	10.0 mL
Vitamin solution	4.0 mL			Vitamin solution	10.0 mL
Reducing agent solution	4.0 mL				
Tungstate solution	0.4 mL				
Resazurin (0.1% solution)	0.4 mL				
Medium preparation: Combine all ingredients except the sodium bicarbonate and the reducing agent into distilled or deionized water, then adjust the total volume to 417.8 mL. Stir the mixture thoroughly, then gently heat it to boiling under an 80 % N₂/20% CO₂ atmosphere. Allow the solution to cool to 45–50 °C before adding the sodium bicarbonate and the reducing agent. Dispense the medium into flasks while maintaining the 80% N₂/20% CO₂ headspace, and sterilize by autoclaving for 15 min at 121 °C (15 psi). After inoculation, replace the headspace gas with an 80% H₂/20% CO₂ mixture.		Medium preparation: Combine all ingredients except the ascorbic acid and thioglycolic acid in distilled water and adjust the total volume to 1 L. Stir the solution thoroughly, then use dilute HCl or NaOH to bring the pH to 7.0-7.5. Just before sterilization, add the thioglycolic acid and ascorbic acid. Dispense the medium into anaerobic culture flasks and autoclave at 121 °C (15 psi) for 15 min.		Medium preparation: Dissolve every component except the vitamin solution in distilled or deionized water and make up the volume to 1 L. Heat gently to boiling while stirring, then allow the mixture to cool to room temperature under a continuous flow of 80% N₂/20% CO₂. Under this inert atmosphere, distribute the medium into culture flasks and autoclave at 110°C (6 psi) for 45 min. Once cooled, and with the vessels held under N₂/CO₂, aseptically add the vitamin solution to complete the medium.	

^*a*^ Source: reference 18.

^*b*^ Weight values are given in grams (g). Volume values (mL) refer to pre-prepared stock solutions of specific concentrations (g L⁻¹). The specified volume is taken to prepare the complete medium. For details on stock solution compositions, see reference 18.

**Fig 1:**
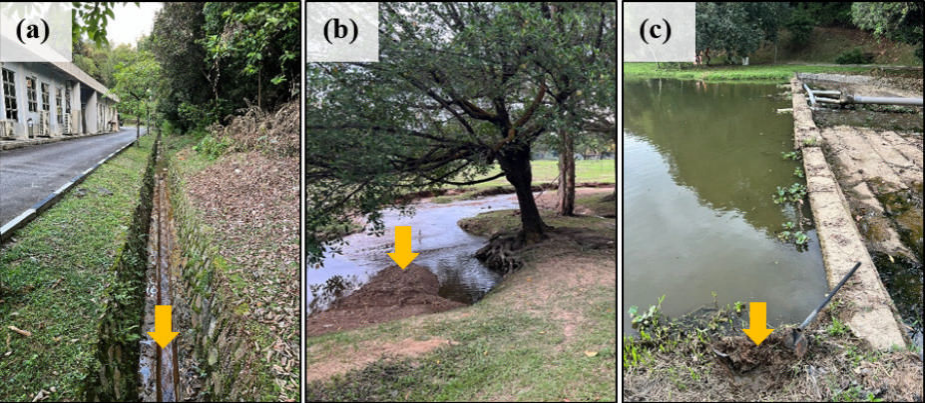
Sample collection points for culture enrichment. Arrows indicate the specific locations where the soil samples were collected.

Inoculum sources were collected from three locations around Tasik Kejuruteraan, UKM, as shown in Fig. 1: (i) at the river inlet into the lake, (ii) along the lake bank, and (iii) near the dam outlet. The selection of these sites was done based on the difference in the organic content and microbial diversity, as these factors influence the microbial community present. Additionally, the environmental conditions, including variations in oxygen levels and nutrient availability, determine the dominance of specific microbial species. For each sample, a 10% (wt/vol) inoculum was added to the serum bottles, which were then purged with N₂ gas to create an anaerobic environment before sealing. The bottles were incubated at 30°C.

The enrichment process involved three consecutive cycles. After each cycle, the growth of target microbial species was assessed, indicated by changes in color and the presence of precipitates in the medium. This ensured the successful enrichment of the desired microbial communities before transferring them into the biosensor setup.

### Electrochemical analysis

A multichannel potentiostat (Quad, Whistonbrook Technologies, UK) was employed to conduct cyclic voltammetry (CV) to evaluate the catalytic activity of the biosensor. Following three stable enrichment cycles, the supernatant of the sample was collected, leaving any precipitate, and transferred to a container fitted with a working electrode (WE), reference electrode (RE), and counter electrode (CE). The electrochemical setup consisted of carbon cloth (CC) as the working electrode, graphite as the counter electrode, and an Ag/AgCl electrode as the reference electrode. CV measurements were performed from −0.6 to +0.2 V versus the standard hydrogen electrode (SHE) at a scan rate of 1 mV/s. At least three scans were conducted, with only the final voltammogram presented in this study; all potentials were referenced to SHE.

### Biosensor setup, enrichment, and operation

**Fig 2:**
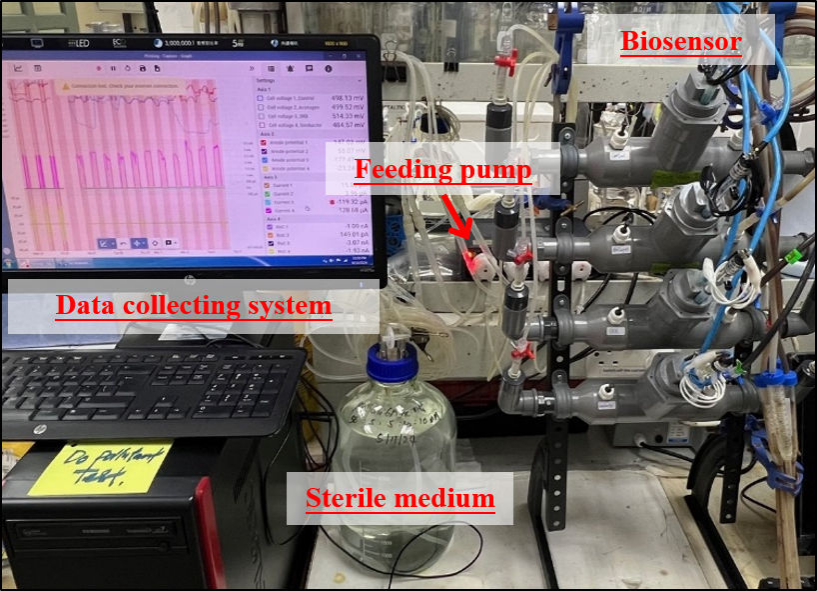
Biosensor setup complete with automated medium feeding and data logging systems.

A customized biosensor system was developed involving automated medium feeding and real-time data collection to ensure the continuous monitoring of microbial electrochemical activity. Figure 2 illustrates the experimental biosensor setup within the laboratory. The biosensor was constructed with an SS304L stainless steel electrode housed in a PVC cap, following the design recorded in Yeo et al. (19). However, the sensor size was increased by 2.5 times to accommodate a larger housing tube, aiming to enhance the surface area for bacterial colonization, increase electrode-bacteria contact, and improve sensor sensitivity. A hole was made at the top of the cap for electrode wiring, which was connected through a modified LAN cable and RJ45 fittings.

The feed medium for the biosensor was prepared in a 5 L laboratory bottle and delivered to each biosensor independently via dosing pumps (Doser 2.4, Jebao, China). Each biosensor was independently infused with 100 mL of fresh medium every day by the pump, unless stated otherwise. The medium comprised 50 mM phosphate buffer solution (PBS) at neutral pH, 5 mM glucose, 5 mM sodium glutamate, 10 mM sodium acetate, 10 mM ammonium chloride, and trace vitamins and minerals. PBS was sterilized by autoclaving at 121°C for 20 min, and the remaining components were added aseptically using a 25 mm, 0.2 µm syringe filter. To eliminate oxygen interference, the medium was then purged with pure nitrogen gas for 15 min to ensure a sterile and anaerobic supply.

The system was built to maintain a controlled flow rate while preventing oxygen contamination, maintaining consistent electrochemical signals across all experiments. This setup played a crucial role in improving the biosensor performance by providing a fixed supply of nutrients while maintaining an optimal environment for microbial activity. Moreover, the automated data collection system is able to significantly provide better tracking of sensor responses to pollutants.

The biosensors were left for a day to test the integrity of the entire operation system. On the following day, the supernatant from the pre-enriched cultures was collected and injected into the four different biosensors marked as control, Acetogen, SRB, and *Geobacter *sp. The biosensors were then left for several days to observe anode potential drops and current increases. If no response was observed, a second inoculum injection was performed to stimulate growth until a response was detected.

### Data capture and alarm setting

Each sensor was independently powered with an applied voltage of 0.5V. An Ag/AgCl reference electrode was positioned next to the anode to measure anode potential. A data logger (ADC-24, Pico Technologies, UK) collected real-time data from the biosensor via a PC, displaying the anode potential and biosensor voltage. Voltage data were converted to current using Ohm’s Law (I = V/R), where I is the current in amperes, V is the sensor voltage, and R is the shunt resistance in line with the anode wire. This setup enabled precise detection of voltage changes. RoC refers to the speed at which the current signal changes over time (µA/min), which is crucial for sudden pollutant events or sensor disturbance detection. The alarm settings were based on the maximum (L_max_) and minimum (L_min_) limits of both current and rate of change (RoC). A stable baseline was established for both parameters, and the alarm limits were set within a specific range. Currently, the limit was typically between 0 and 1.5 mA, whereas for ROC, it was between −10 µA/min and +10µA/min. When either current or RoC values exceeded their respective thresholds, an alarm would be automatically triggered by the system on the PC, alerting the operator.

### 4-Nitrophenol toxicant test and biosensor recovery

A 4 NP solution was prepared by diluting 10 g of 4 NP in 100 mL of deionized water. For the test, 20 mL of the solution was injected directly into each biosensor housing, followed by a 2 h wait before another 20 mL injection. The same procedure was repeated the next day after leaving the biosensors undisturbed overnight (~8 h). On the third day, biosensor recovery was initiated by adding 100 mL of deionized water, followed by another 100 mL after 4 h. The biosensors were then left untouched for further recovery without additional testing.

### Liquid sample analysis

Approximately 10 mL of sample was collected following pollutant injection during the pollutant tests. The samples were filtered using 0.45 µm syringe filters before the analysis. pH and conductivity measurements were recorded with a benchtop multiparameter pH meter (HI2020-02 Edge, Hanna Instruments, Malaysia) equipped with pH and conductivity probes. Chemical oxygen demand (COD) and total nitrogen−ammonium (N−NH4^+^) levels were measured using COD and total nitrogen reagent kits (Hach, USA), and these values were correlated with the biosensor signal data.

### Microbial community analysis

The pre-colonized anode community analysis was not performed because the enriched medium was specifically designed to target certain bacterial groups known for their electrochemical functionality. Using the biosensor as a control without pre-enrichment would require an extended incubation period of several weeks and would likely result in minimal electrochemical activity, making it impractical for comparative analysis. Moreover, pre-colonized anodes initiated from fresh cultures would not provide a controlled baseline for assessing electrochemical performance. This approach aligns with common practices in microbial electrochemical studies, where enrichment enhances reproducibility and ensures functional relevance.

The samples were submitted to Patriot Biotech Sdn. Bhd., a local laboratory, for further processing. This included DNA extraction, PCR amplification, and validation, followed by amplicon-based compositional analysis targeting Bacteria/Archaea (16S) using the sequenced DNA. The DNA was extracted using a commercial DNA extraction kit, following the manufacturer’s protocol optimized for environmental biofilm samples. The V3–V4 region of the 16S rRNA gene was amplified using standard primers through PCR prior to sequencing on the Illumina MiSeq platform.

The post-sequencing report included a detailed review of bacterial abundance, providing insights into the microbial composition. A relative abundance network analysis was conducted to show the correlation between the natural microbial community, pre-enriched cultures, and biosensor-enriched microbial communities. These results are presented in the final section of the results and discussion.

## RESULTS AND DISCUSSION

### Enrichment of microbial cultures in serum bottles

Enrichment was focused on *Geobacter* sp., sulfate-reducing bacteria (SRB), and acetogens due to their well-established roles in microbial electrochemical systems. These microbial groups have also demonstrated significant biotechnological applications in wastewater treatment and bioremediation, as supported by several studies (20, 21). *Geobacter* is well-known for its ability to perform direct extracellular electron transfer (EET) via c-type cytochromes and conductive pili, making it efficient for anodic biofilm formation and current production. SRBs involve biocathode activity by reducing sulfate and participating in electron transfer. On the other hand, acetogens were chosen specifically for their role in producing acetate, a key electron donor that supports the metabolism of both *Geobacter* and SRBs, thus improving electron flow within mixed microbial communities (22, 23).

**Fig 3:**
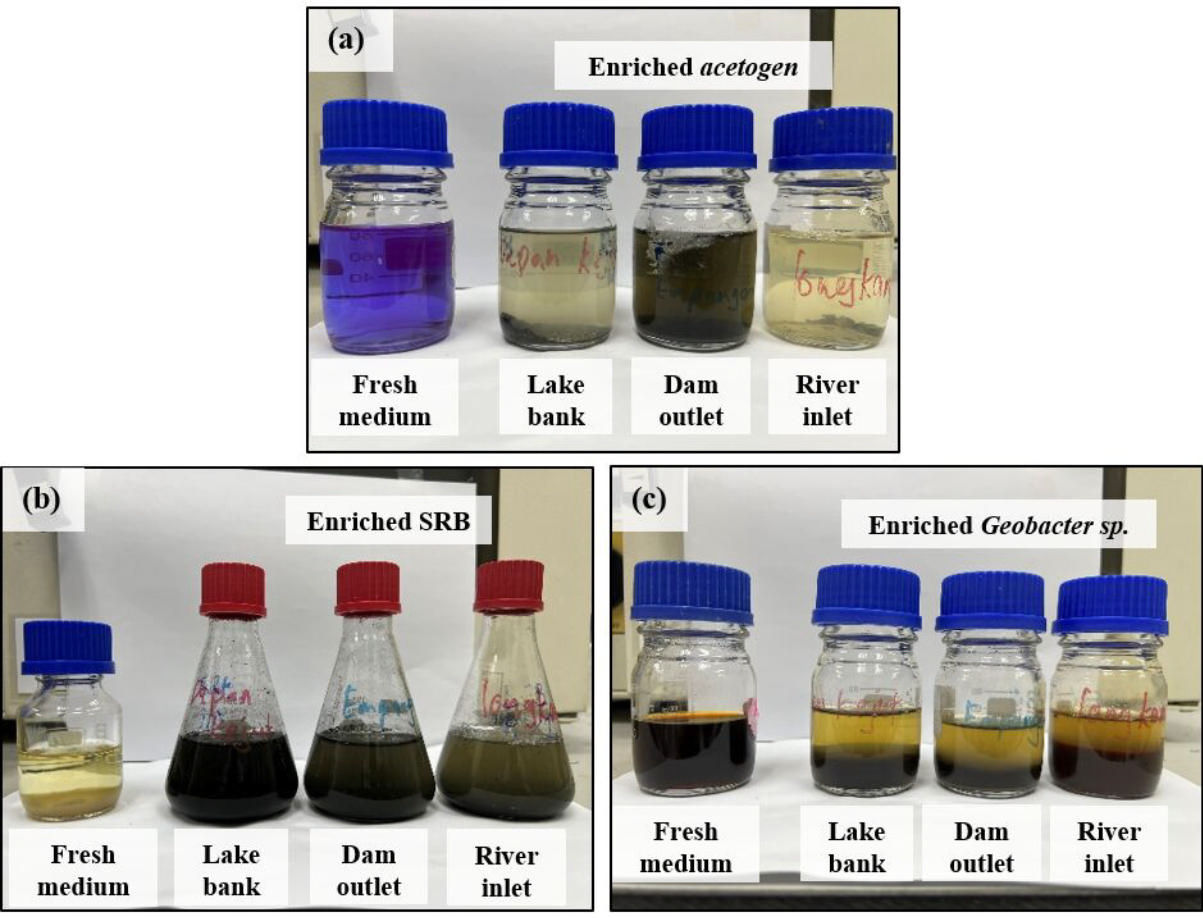
Successfully enriched microbial cultures, showcasing acetogen, sulfate-reducing bacteria, and* Geobacter* species isolated from three distinct locations near the lake.

Figure 3 illustrates the enriched microbial cultures obtained from the area around Tasek Kejuruteraan. Each medium contained specific ingredients designed to indicate the metabolic activities of targeted microbes in the culture. Although the enriched microbes appear dominant, the presence of other bacterial species thriving in the media cannot be disregarded. A more detailed community analysis is required to confirm the specific species and any co-existing microbial populations in the enriched cultures.

In the acetogen medium, resazurin served as an indicator of acetogenic activity. The color change from blue-pink to clear confirmed active anaerobic growth, as the acetogen consumed all available oxygen within the container. This metabolic mechanism indicates that acetogens were actively fermenting substrates to produce acetate, the main process in anaerobic respiration. Notably, the microbial culture collected from the lake bank and river inlet was observed to be clearer, suggesting a higher dominance of acetogenic bacteria in these environments compared with the dam outlet sample, which retained a more cloudy appearance (Fig. 3a).

For sulfate-reducing bacteria (SRB), their presence was indicated by the blackening of the medium due to the formation of FeS. Ferrous sulfate (FeSO₄) was added to the medium as a sulfate promoter, with iron acting as an indicator for SRB activity (Fig. 3b). The formation of the FeS precipitate across all cultures proves that SRB bacteria had effectively utilized sulfate as an electron acceptor, showing an active sulfate reduction within the enriched microbial community. The intensity of the black precipitate formed varied among the samples.

For iron-reducing bacteria (*Geobacter *sp.), the reduction of Fe(III) to Fe(II) was evident through the formation of dense precipitates, which settled at the bottom of the container. The lake bank and river inlet samples demonstrated a higher Fe(II) formation, as can be observed by the larger sedimentation compared with the dam outlet sample, which exhibited less precipitate formation. This confirmed the activity of iron-reducing microbes in the medium (Fig. 3c).

### Electrochemical activities of the enriched cultures

**Fig 4:**
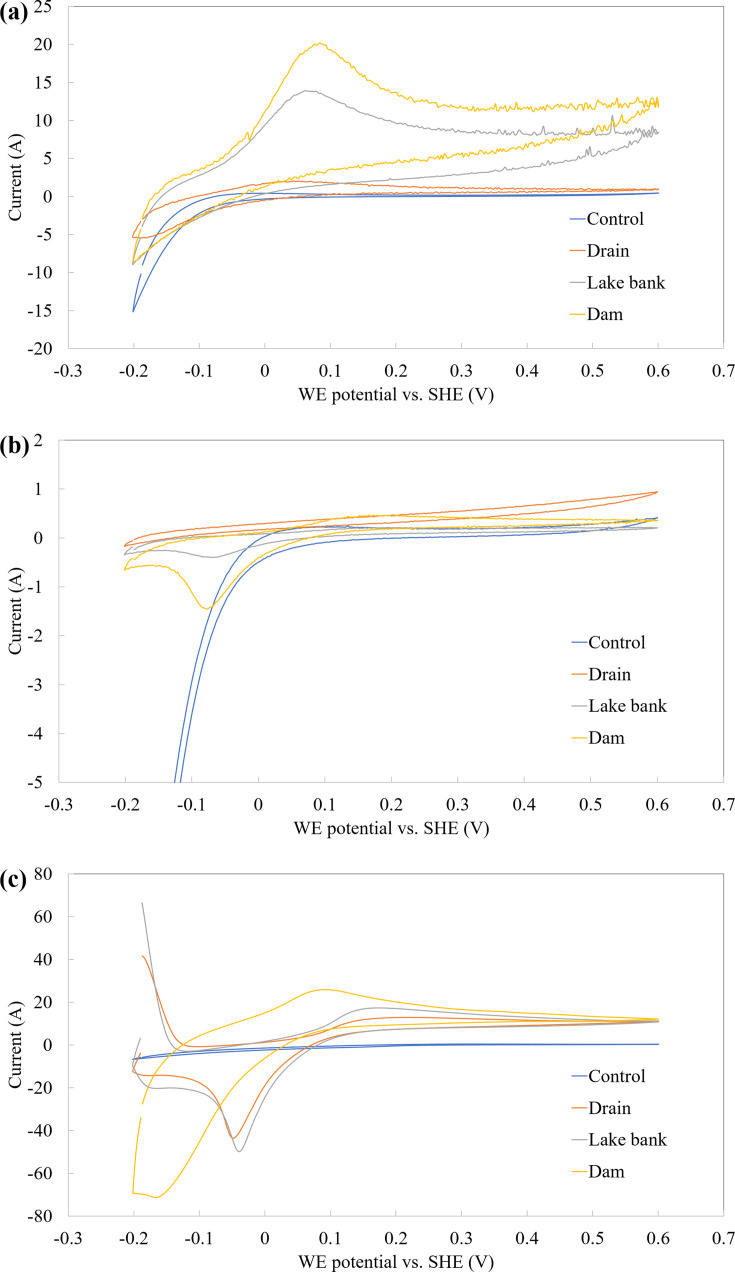
Third scan of cyclic voltammograms of enriched microbial cultures grown in specific media: (a) acetogen, (b) sulfate-reducing bacteria (SRB), and (c) *Geobacter *sp. For comparison, the control represents fresh medium without microorganisms.

Figure 4 illustrates the cyclic voltammetry (CV) analysis of enriched microbial cultures obtained from three different sampling locations: the inlet drain entering the lake, the lake bank, and the dam site before the outlet. The cultures were enriched using three specific media targeting acetogen, sulfate-reducing bacteria (SRB), and *Geobacter *sp. The enrichment periods varied, with acetogen and SRB cultures being incubated for 1-2 days and *Geobacter* cultures requiring up to 3 days to exhibit activity before analysis. 

Cyclic voltammetry (CV) analysis was performed to evaluate the electrochemical activity of the enriched microbial cultures, ensuring stabilization over multiple cycles. The first and second CV scans (data not shown) showed fluctuations in the peak intensities, suggesting that the microbial electrochemical system was still stabilizing. Fluctuations are common in these systems, as to achieve a steady-state electron-transfer process, a few cycles are required for the electroactive microbes. Thus, the third scan CV (Fig. 4a through c) was selected as the representative data since it demonstrated consistent and stable peak conditions, indicating the electron transfer mechanisms had fully developed. The initial CV scans had been included in SI as a reference to illustrate the transition of the system towards stability and consistency.

In the CV results for acetogen-enriched cultures (Fig. 4a), a prominent oxidation peak at approximately +0.1 V was observed within the scan range of −0.2 to +0.6 V. This peak is likely attributed to substrate oxidation facilitated by the microbial community. The inoculum from the dam site exhibited the highest oxidation peak, followed by the lake bank sample, whereas the drain site sample showed only a minor peak. This discrepancy could be due to the anoxic environment at the drain site, which is less favorable for aerobic microorganisms typically associated with acetogens. Notably, no oxidation peak was detected in the control medium, indicating the absence of electrochemical activity without microbial involvement.

Furthermore, this activity may be linked to indirect electron transfer mechanisms, as frequently noted in the literature, particularly in processes involving the Wood–Ljungdahl pathway of CO_2_ reduction. This suggests that the microbial species in question require external reducing power to support the reduction process, further underscoring the complexity of microbial interactions and electron transfer pathways in such systems (24, 25).

The CV results for SRB-enriched cultures (Fig. 4b) showed a minimal reduction peak at approximately −0.1 V for the dam site sample, with a slightly smaller peak observed for the lake bank sample. No significant peaks were detected for the drain site sample. This trend suggests that SRB species in the studied locations predominantly thrive in anaerobic environments, explaining their limited presence in the oxygenated conditions at the drain site. The control medium exhibited a reduction curve near −0.2 V, possibly caused by active compounds like ascorbic acid or thioglycolic acid added to the medium to create a low-oxygen environment conducive to SRB growth. The species has previously been identified as playing a crucial role in facilitating hydrogen production within the biocathodes of microbial electrolysis cells and electrosynthesis systems, highlighting its significance in the electrochemical field. This species likely possesses a *c*-type cytochrome network, potentially located in the periplasm, along with associated periplasmic hydrogenases and transmembrane complexes. These molecular components not only contribute to heavy metal reduction in wastewater treatment but also support the H_2_ cycle and production when an external reducing power is applied electrochemically, typically up to −1.0 V, as suggested by recent studies. These findings underscore the versatile applications of the species in both environmental remediation and renewable energy systems (26–28).

For *Geobacter*-enriched cultures (Fig. 4c), both oxidation (+0.1 V) and reduction (−0.05 V) peaks were observed, aligning with the electrochemical behavior typically associated with *Geobacter *sp. This microorganism is well-known for its role in bioelectrochemical systems, such as microbial fuel cells (MFCs) and microbial electrochemical technologies (METs), where it acts as a bioanode catalyst in wastewater treatment (29–31). Similar to the other media, the samples from the dam site exhibited the highest electrochemical activity, followed by the lake bank and drain site samples. The control medium showed no notable activity, confirming the role of *Geobacter* in facilitating the observed electrochemical behavior.

The CV analysis highlights distinct electrochemical activities associated with each microbial group, influenced by the environmental conditions of the sampling locations. The dam site consistently demonstrated higher activity, likely due to its favorable conditions for microbial growth, whereas the drain site showed limited activity due to its oxygenated environment. These results provide valuable insights into the electrochemical behavior of enriched microbial cultures and their potential applications in bioelectrochemical systems.

### Post-enrichment and toxicity testing of microbial biosensors

**Fig 5:**
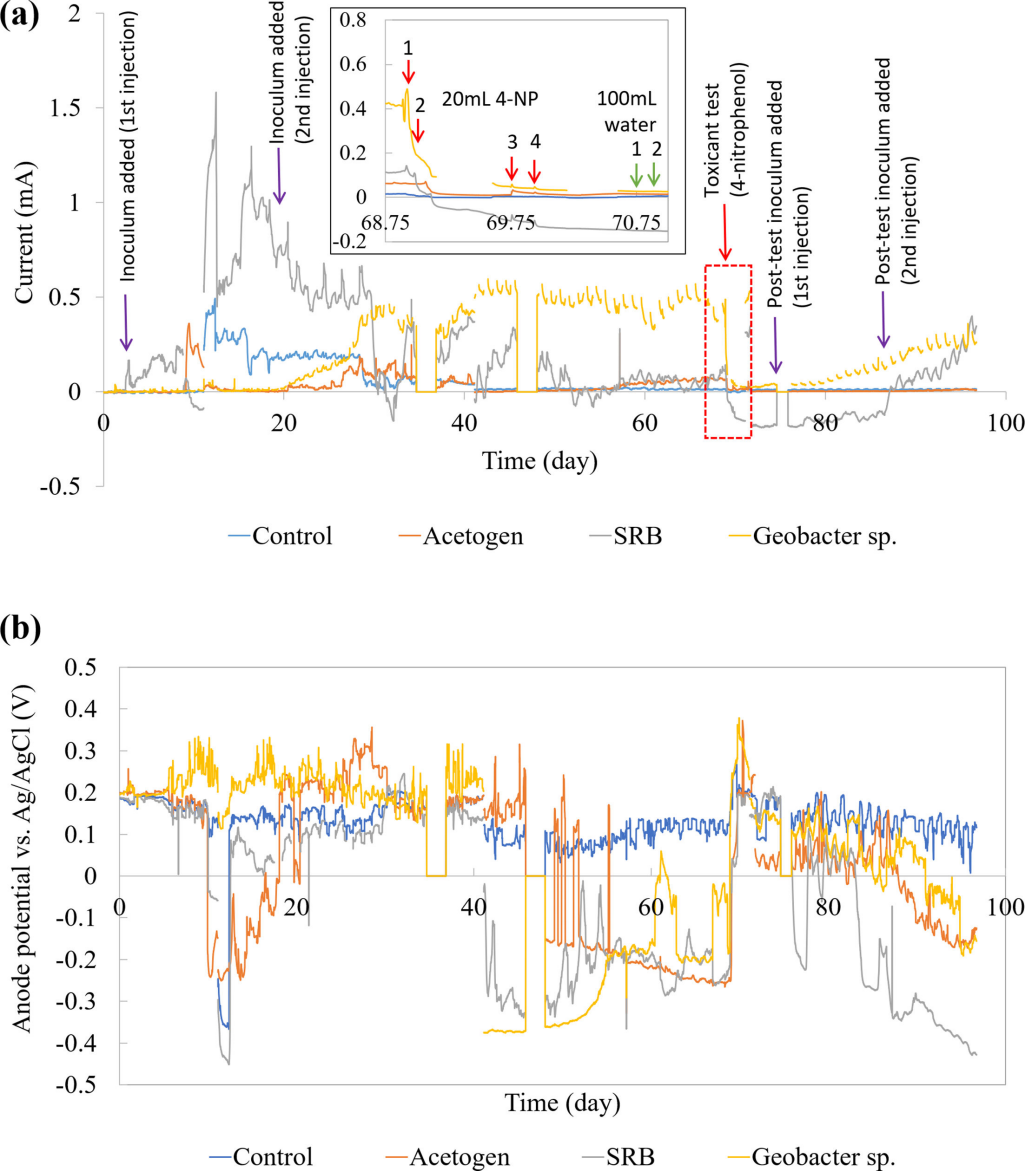
(a) Current and (b) anode potential profiles of biosensors enriched with pre-enriched cultures compared with control.

Figure 5 illustrates the current and anode potential profiles of biosensors enriched with pre-enriched microbial cultures, compared with a control group that was directly inoculated with natural samples without undergoing pre-enrichment. The performance of each biosensor was evaluated based on the current response and sensitivity to pollutant exposure.

From the current profile (Fig. 5a), the first inoculum injection immediately stimulated the growth of the SRB (sulfate-reducing bacteria) biosensor, followed by the control. The *Geobacter*-enriched biosensor showed a delayed response, with significant current increases occurring only after the second injection, alongside the acetogen-enriched biosensor. This observation suggests that although *Geobacter* is electrochemically active, it does not readily adapt to biosensor conditions and requires time to establish growth (29, 30). This delayed adaptation is likely due to *Geobacter*’s dependence on extracellular electron transfer mechanisms that require the development of stable biofilms on the electrode surface before peak performance can be achieved. In contrast, the SRB-enriched biosensor provided an immediate response, potentially due to residual FeS or other by-products from the pre-enrichment medium. FeS is a common by-product in SRB activity, functioning as a catalyst to enhance electron transfer. Besides, the rapid response observed in SRB-enriched biosensors may also indicate that their metabolic pathways are suitable for initiating electrochemical activity. However, these hypotheses warrant further investigation through techniques such as CV and electrochemical impedance spectroscopy (EIS) to confirm the phenomenon.

After stable current profiles were achieved (approximately 30 days), the biosensors were considered stable under the test conditions. A toxicant test was performed around day 68.8, as shown in the inset of Fig. 3a. Among all the biosensors, the *Geobacter*-enriched biosensor elicited the most significant current drop, declining sharply, from 0.478 mA to 0.093 mA within a single day after 4 NP exposure. In comparison, the SRB-enriched biosensor also exhibited a response, but the current drop was less significant, declining from 0.190 mA to 0.094 mA. On the other hand, the acetogen-enriched and control biosensors displayed minimal changes in current, indicating their limited electrochemical performance. The sharp decline highlights that *Geobacter*’s high sensitivity to toxicants makes it a potential candidate in electrochemical sensors for rapid pollutant detection. Additionally, the rapid response highlights the high sensitivity of *Geobacter*-enriched biosensors and is likely due to the presence of mature electroactive biofilms with well-developed extracellular electron transfer (EET) pathways. The control biosensor showed no significant response to 4 NP exposure. In comparison to the previous study by (32, 32), 4 NP as a toxicant model showed instant response as in this study.

Besides, another important observation from Fig. 5a is the recovery of the biosensors following toxicant exposure. The recovery process started around day 76.5 for both *Geobacter* and SRB-enriched biosensors, approximately 8 days after pollutant exposure. However, the *Geobacter*-enriched biosensor exhibited much faster recovery, quickly generating higher current following the 1_st_ post-test inoculum injection. SRB took longer to recover, with an increase in current only after the 2nd post-test inoculum injection. In contrast, the acetogen and control biosensors do not show any noticeable recovery, as their signals remained minimal throughout the pollutant testing. The rapid recovery response by *Geobacter* is likely attributed to their efficient EET pathways, which facilitate the quick restoration of their metabolic activity.

*Geobacter* species are well-established as key players in microbial electrochemistry, being responsible for substrate oxidation and electricity generation, which explains their direct response to toxicants (33). Generally, *Geobacter* species consist of a complex chain reaction to transfer electrons from intracellular carriers to extracellular minerals, a process mediated by *c*-type cytochromes and conductive pili (34, 35). Their ability to form pili-like structures assists in efficient electron transfer, enhancing their suitability for biosensing applications. Meanwhile, SRB are more commonly associated with biocathodes, where they support hydrogen production and carbon dioxide reduction in microbial electrolysis cells (MECs) and microbial electrosynthesis cells (MESCs) (26, 36). This suggests that SRB growth may have occurred predominantly on the cathode side of the sensor rather than the anode, a hypothesis requiring further validation.

In contrast, acetogen-enriched cultures showed lower performance in this experiment. Literature indicates that acetogens are not inherently electrochemically active but instead contribute through the Wood–Ljungdahl pathway, which supports indirect electron transfer at biocathodes (1). They reduce CO₂ by consuming hydrogen produced from abiotic cathodic proton reduction, potentially explaining their limited activity in this test.

The control biosensor displayed the lowest performance, with minimal current changes and a stagnant anode potential throughout the 100-day experiment. This highlights the critical importance of pre-enrichment in enhancing biosensor viability, significantly reducing startup times from weeks to mere days.

**Fig 6:**
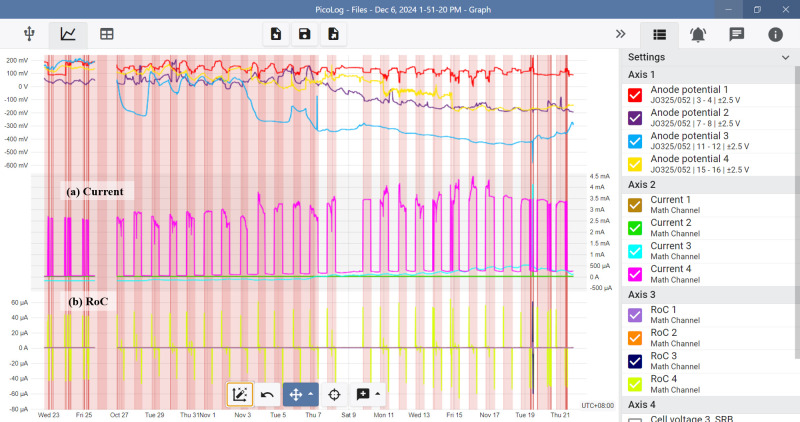
The data logger platform displaying real-time data from its dedicated cloud interface. The current (represented by the red line) was simulated and captured, with spikes in the rate of change (RoC) graph detected by the software application. These spikes triggered notifications for off-limit conditions and irregularities.

Figure 6 presents data from the data logger platform, which monitors both the current and the rate of change (RoC) as key parameters. A non-compliance signal was intentionally simulated (red line) on the current graph by pumping an excessive amount of medium from the stock bottle over 10 min. The high concentration of the periodic stock medium produced repeated peak signals, as shown in Fig. 6a.

The operational limits of the biosensor, as reflected by the current curve within the 0–1.5 mA range, indicate its optimal health. However, these parameters alone are insufficient to detect signal non-compliance. Instead, the RoC metric plays a pivotal role by measuring the rate at which the signal changes, even within the predefined operational limits of ±10 µA/min. These maximum and minimum RoC values were determined to be adequate for the biosensor in this study. Baseline data recorded during the biosensor’s stable and optimal operation provide a critical reference for setting these RoC thresholds.

Figure 6b highlights the RoC profile (yellow line), demonstrating signal changes caused by pumping excessive medium from the stock bottle. This analysis underscores the importance of combining current and RoC metrics for effective biosensor monitoring and identifying anomalies.

### Microbial diversity on biosensor surfaces

**Fig 7:**
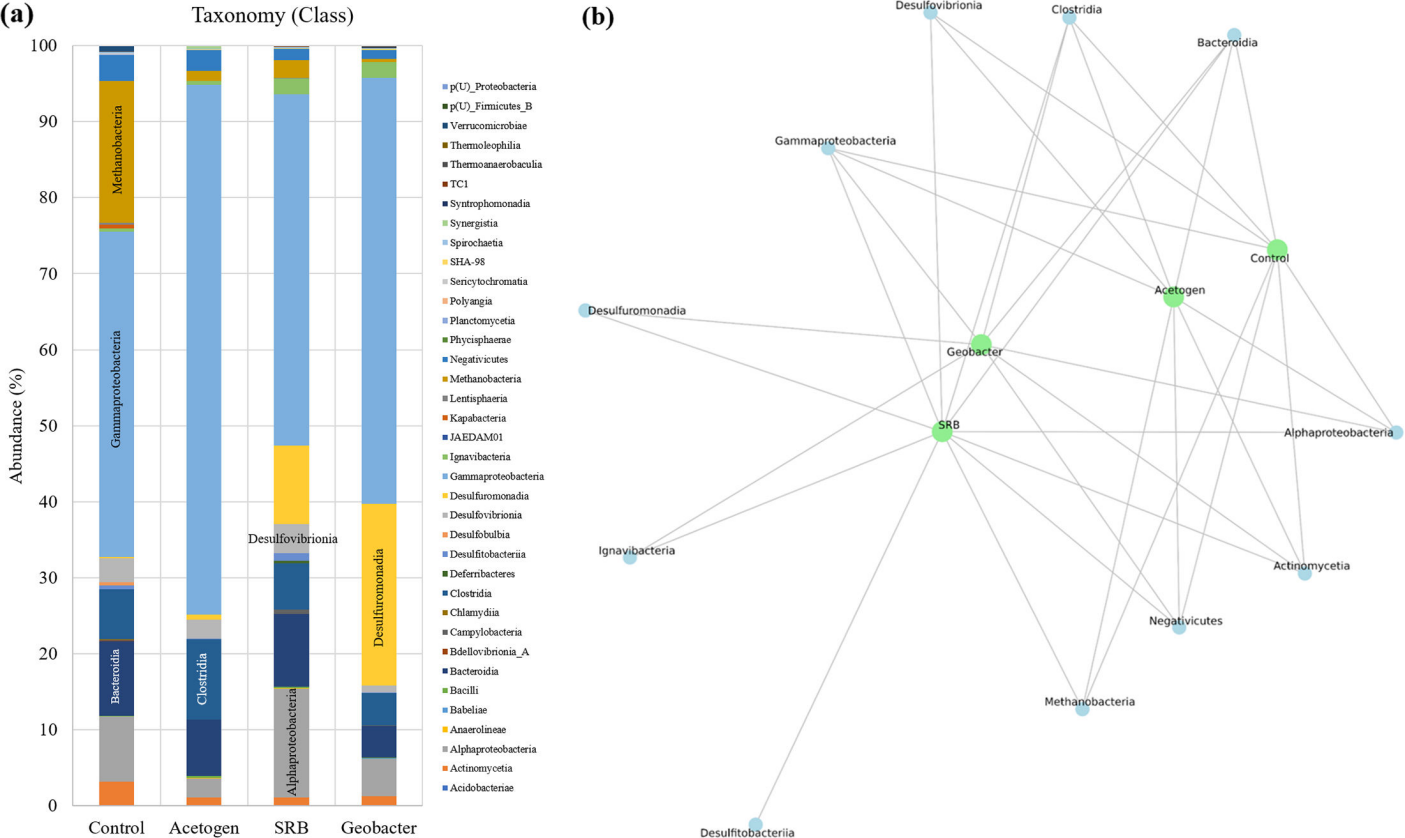
(a) Microbial taxonomy and (b) bipartite network at the class level, illustrating the diversity and abundance on the biosensor anode surface after achieving a stable current. The names on the X-axis and green dots represent those used in the pre-enriched cultures, which were subsequently used to re-enrich the microbial community on the biosensors.

Figure 7 presents the microbial taxonomy and bipartite network at the class level, analyzing the microbial community sampled from the anode surfaces of biosensors. The pre-colonized anode community analysis was not performed because the enriched medium was specifically designed to target certain bacterial groups known for their electrochemical functionality. Using the biosensor as a control without pre-enrichment would require an extended incubation period of several weeks and would likely result in minimal electrochemical activity, making it impractical for comparative analysis. Moreover, pre-colonized anodes initiated from fresh cultures would not provide meaningful data for assessing electrochemical performance.

The taxonomy data demonstrated in Fig. 7a highlight the relative abundance of different microbial classes across the enriched and control biosensor samples, whereas Fig. 7b presents the microbial interactions in a bipartite network analysis. The most prominent observation (Fig. 7a) is the dominance of *Gammaproteobacteria*, which accounts for 40%–70% of the total microbial community across all four samples. *Gammaproteobacteria* are vital in marine, coastal, and land ecosystems, serving as key players in nutrient cycling processes. The high abundance of *Gammaproteobacteria* indicates that these microbes contribute to the degradation of organic compounds, which are important for biosensor function. At the lower taxonomic level, the orders *Burkholderiales* and *Enterobacterales* were the most abundant within this class. These microbes, being gram-negative and facultatively anaerobic, create a strictly anaerobic environment conducive to biosensor operation while supporting the growth of electrochemically active microbes through symbiotic interactions. Similar observations have been reported across various microbial electrochemical systems. For example, Spurr et al. (37) demonstrated in their study involving multistage microbial fuel cell (MFC) sensors that microbial communities from anodic biofilms and non-electrode-associated biofilms generate biosensor signals through syntrophic interactions between fermenting bacteria and electrogens. In another related study, Lim et al. (25) investigated hydrogen-producing biocathodes and found that Proteobacteria were the most abundant community members, playing a crucial role in maintaining strict anaerobic conditions essential for optimal system performance.

Beyond *Gammaproteobacteria*, a notable proportion of *Methanobacteria* (a class of archaea) was observed in the control sample, representing nearly 19% of the microbial population. This contrasts sharply with other samples, where *Methanobacteria* constituted less than 3%. These archaea were likely present due to their dominance in the natural environment from which the original inoculum was derived. However, when examining the bipartite network (Fig. 7b), methanogens appear to be irrelevant to the *Geobacter* samples. This observation suggests that the process of enrichment, followed by application to the biosensor, emerges as a promising approach to enhance the biosensor’s performance by fostering the right functional microbial community. Methanogens can hinder the performance of microbial fuel cell (MFC) systems by reducing electron generation efficiency and negatively impacting the overall system (38). These microorganisms consume substrates intended for electrogenic bacteria and do not produce detectable electrical signals, making their presence challenging to monitor. Encouragingly, methanogens are consistently suppressed in *Geobacter-*rich dominant environments, a phenomenon demonstrated and supported by the findings of Dzofou et al. (31). This microbial selection was able to enhance current generation, as *Geobacter* spp. depend on extracellular electron transfer (EET) to directly donate electrons to the anode and improve biosensor sensitivity. Moreover, this suppression highlights the potential of *Geobacter-*enriched systems in mitigating methanogenic interference, thereby enhancing the efficiency and stability of microbial electrochemical systems. This study also focuses on examining methanogens and their interactions with other microbial communities and identifying strategies to mitigate their impact on system performance.

Other studies highlight the role of *Proteobacteria* in supporting electrochemical reactions in biosensors or microbial fuel cells. Although direct involvement in electrochemical pathways has been attributed to some species, our findings suggest that *Proteobacteria*, especially *Gammaproteobacteria*, play a crucial indirect role. As an abundant environmental group, they contribute to the breakdown of complex organic compounds into simpler molecules (39). This metabolic activity facilitates the growth of anaerobic chemoautotrophic bacteria, such as iron-reducing and sulfate-reducing bacteria, which rely on short-chain volatile fatty acids like acetate and lactate as substrates.

By breaking down complex sugars and other organics into accessible intermediates, *Gammaproteobacteria* and related *Proteobacteria* indirectly support the electrochemical activity essential for biosensor functionality. This highlights their importance in creating a synergistic microbial ecosystem on the anode surface, optimizing conditions for electricity generation. These findings reinforce the importance of pre-enrichment strategies in building the microbial community composition to enhance biosensor functionality.

### Conclusion

**Fig 8:**
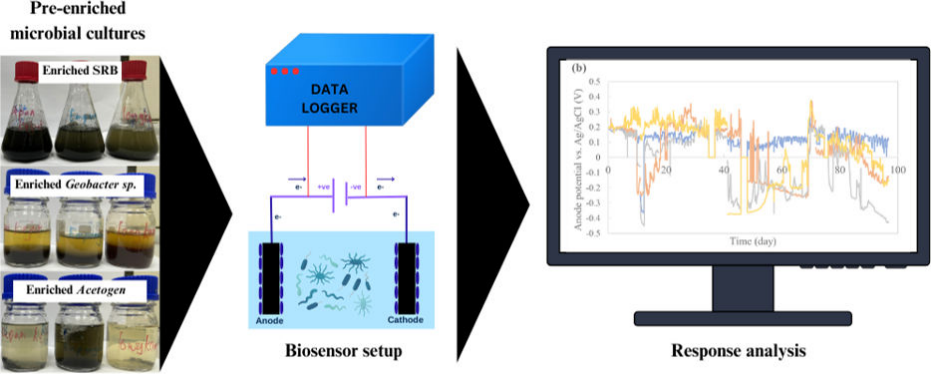
Pre-enriched microbial communities—sulfate-reducing bacteria (SRB),* Geobacter *spp., and acetogens—were selectively grown in defined media and transferred to tubular biosensors. Following chronoamperometric conditioning to a stable plateau in current and anode potential (under 20  days), their electrochemical signatures were logged. *Geobacter*-enriched sensors exhibited the strongest anodic currents; SRB-enriched sensors produced cathodic (negative) signals; acetogen-enriched sensors showed slower, lower-intensity responses; and control sensors inoculated with natural microbiota displayed minimal activity. This workflow underscores how targeted pre-colonization enhances both sensitivity and durability in microbial electrochemical water-quality sensors.

This study highlights the importance of pre-colonized microbial cultures in enhancing microbial electrochemical sensor performance for water quality monitoring. Targeted enrichment of *Geobacter* species, sulfate-reducing bacteria (SRB), and acetogen microbes significantly improved detection sensitivity and response time compared with natural inoculum controls (Fig. 8). *Geobacter*-enriched sensors demonstrated superior electron transfer and pollutant detection, whereas SRB and acetogen sensors showed distinct but less efficient electrochemical activity. Pre-enrichment shortened startup time and ensured stable operation, emphasizing its critical role in optimizing biosensor functionality for environmental applications, particularly in addressing water pollution and promoting sustainable monitoring technologies. Future work should focus on the long-term stability of enriched microbial communities and their specificity toward different types of pollutants to ensure advancement of these systems toward commercialization. Moreover, studies should be conducted on scaling up the technology for real-time applications in industrial and municipal wastewater treatment settings.

### Synopsis

Pre-colonization with targeted microbial cultures enhances microbial electrochemical sensor performance, offering improved sensitivity and robustness for water quality monitoring applications.

## Data Availability

The microbial diversity profiles generated from biosensor surfaces in this study have been deposited in the National Center for Biotechnology Information (NCBI) and are publicly accessible at: https://www.ncbi.nlm.nih.gov/sra/PRJNA1294856.
